# HOME CARE DYADS AND STAKEHOLDERS IN THE CONVOY OF SOCIAL RELATIONSHIPS: A BASIS OF COMMUNITY-BASED RCT

**DOI:** 10.1093/geroni/igad104.1805

**Published:** 2023-12-21

**Authors:** Elise Hu, Lijuan Yin, Maria Caceres

**Affiliations:** University of Illinois Chicago School of Public Health, Chicago, Illinois, United States; University of Illinois Chicago, Chicago, Illinois, United States; University of Illinois Chicago, Chicago, Illinois, United States

## Abstract

Relationships within homecare dyads are the core in both informal and formal caregiving. Home care aides (HCA), a type of formal (paid) caregivers, provide in-home non-medical care to older adults who need assistance with daily activities. The Pro-Home study relied on client-HCA relationships and collaborating homecare stakeholders to recruit dyads in this randomized controlled trial (Randomized N = 128). Participating clients were mostly unmarried and unable to meet with family/friends multiple times a week. More than half lived alone, two thirds were cared for by a HCA who was not a family member, and the majority reported having a good relationship with their HCA. Most participating clients kept the same HCA throughout the 4-month intervention, before and throughout the ongoing COVID-19 pandemic. These characteristics highlight the critical role of homecare workers in caring and health promotion as older clients’ social convoy. However, the pandemic has posed challenges to these dyads. A higher proportion of clients participating in the intervention during the pandemic lost or were reassigned a HCA (20%) compared to those completed the intervention prior to the pandemic (10%). Our community collaborators reported increased staff turnovers and nationwide HCA shortages that created difficulties in care coordination. These findings will inform the ongoing debate on future financing and delivery systems of long-term services and supports, which are increasingly provided in home and community settings in the U.S. and other countries.

